# Nonketotic hyperglycemia hemichorea and hemiballismus: a case report

**DOI:** 10.1186/s13256-023-04332-y

**Published:** 2024-03-12

**Authors:** Abhishek Dixit

**Affiliations:** Department of Neurology, Institute of Medical Science, Banaras Hindu University, Varanasi, India

**Keywords:** Diabetic striatopathy, Hyperglycemic hemichorea-hemiballismus, Nonketotic hyperglycemia, Type 2 diabetes mellitus, Putaminal hyperdensity, Neurotransmitter alterations, Acute choreiform movements

## Abstract

**Background:**

Diabetic striatopathy, also known as hyperglycemic hemichorea-hemiballismus, is a rare movement disorder associated with nonketotic hyperglycemia in patients with poorly controlled diabetes mellitus. The pathophysiology is not fully elucidated but may involve hyperviscosity, ischemia, and alterations in basal ganglia neurotransmitters.

**Case presentation:**

We present a case of a 64-year-old Asian female patient with longstanding poorly controlled type 2 diabetes mellitus who developed abrupt-onset right-sided hemichorea-hemiballismus. Laboratory results showed hyperglycemia without ketoacidosis. Neuroimaging revealed left putaminal hyperdensity on computed tomography and T1 hyperintensity on magnetic resonance imaging. With insulin therapy and tetrabenazine, her movements improved but persisted at 1-month follow-up.

**Discussion:**

This case illustrates the typical features of diabetic striatopathy, including acute choreiform movements contralateral to neuroimaging abnormalities in the setting of nonketotic hyperglycemia. While neuroleptics may provide symptomatic relief, prompt glycemic control is critical given the risk of recurrence despite imaging normalization.

**Conclusion:**

Diabetic striatopathy should be recognized as a rare disorder that can occur with poorly controlled diabetes. Further study of its pathophysiological mechanisms is needed to better guide management. Maintaining tight glycemic control is essential to prevent recurrence of this debilitating movement disorder.

## Introduction

Diabetic striatopathy, also known as hyperglycemic hemichorea-hemiballismus, is an uncommon movement disorder occurring in patients with poorly controlled diabetes and hyperglycemia without ketoacidosis. The estimated prevalence is around 1 per 100,000 diabetic patients, most commonly elderly Asian women with longstanding diabetes [1]. The pathophysiology is unclear, but proposed mechanisms include hyperviscosity-induced ischemic damage, alterations in neurotransmitters such as gamma-aminobutyric acid, and blood–brain barrier dysfunction [2]. Patients typically present with hemichorea-hemiballismus affecting one side of the body. Neuroimaging classically reveals unilateral hyperdensity on computed tomography (CT) or T1 hyperintensity on magnetic resonance imaging (MRI) in the basal ganglia, which can resolve after correcting hyperglycemia [1, 3]. The involuntary movements often improve within weeks of optimizing glycemic control, but anti-dopaminergic medications may also be needed [2]. Recurrence can occur in approximately 18% of cases despite imaging normalization [1]. Here we present a case of diabetic striatopathy in an elderly female patient with poorly controlled diabetes who developed acute right-sided hemichorea-hemiballismus and elevated blood glucose without ketoacidosis. We review the characteristic features of this uncommon movement disorder and discuss the proposed pathophysiological mechanisms and treatment approaches.

## Case report

The patient is a 64-year-old Asian woman who was brought to the emergency department (ED) with uncontrollable, spontaneous right upper limb and right lower limb movements that started 3 days prior to admission and a history of poorly managed type 2 diabetes mellitus. Initially she had involvement of the right hand; these movements eventually included the complete right arm and the right foot as well. The patient’s right upper limb had pain, but other symptoms were absent. She denied any prior instances of such uncontrollable motions, as well as any viral infections or wounds that had recently occurred. In addition, she denied any other symptoms including headaches, chills, fever, or chest pain. Her vital signs were also normal.

**Fig. 1:**
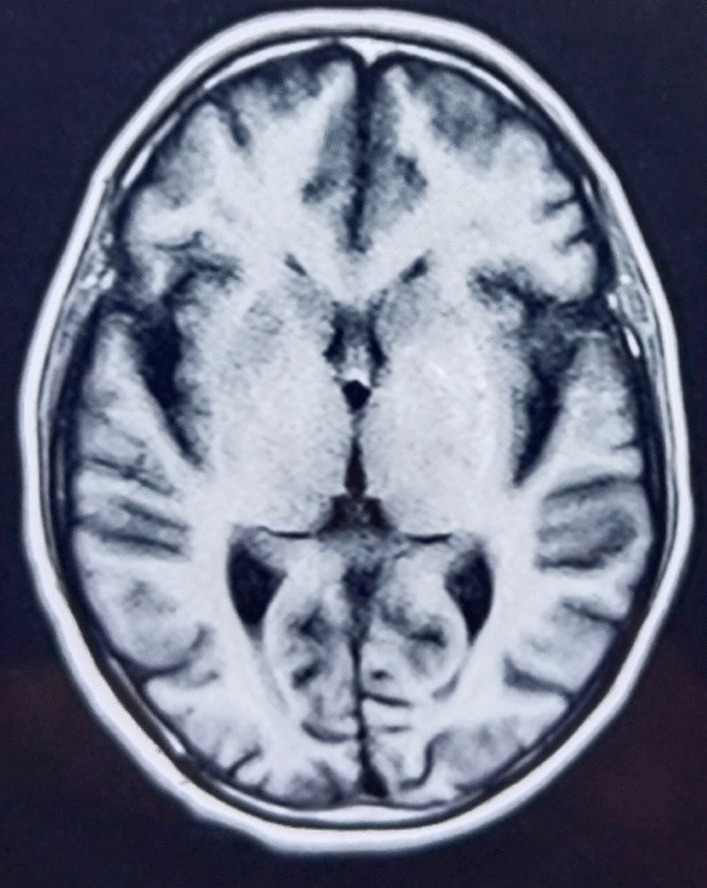
Hypointensity in the left lentiform on T1 nucleus

**Fig. 2:**
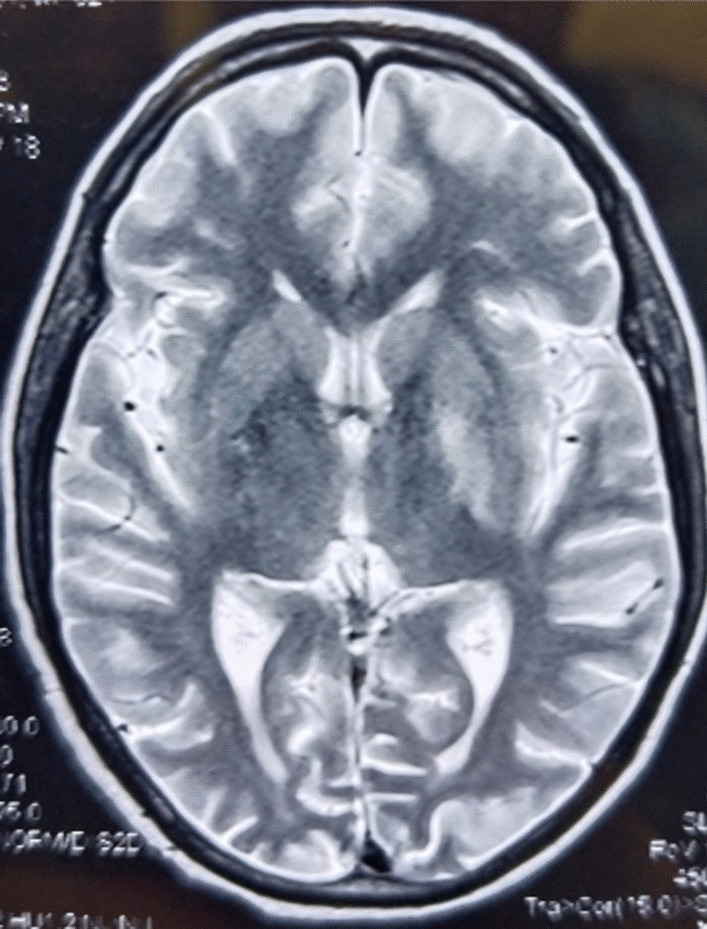
Hyperintensity in left lentiform nucleus on T2

Upon examination, writhing movements were seen across the entire right upper limb. Muscle tension by patient allowed the her to voluntarily stop these motions although it was extremely painful and spread from the shoulder to the hand. From the feet to the knees, both lower limbs showed decreased sensitivity to mild touch (video 1). The patient had symmetrical limb strength and no cranial nerve impairments or abnormal gait patterns. The rest of the examination was uneventful. Initial lab results showed elevated levels of blood urea nitrogen (30 mg/dL), creatinine (1.52 mg/dL), and serum glucose (423 mg/dL). Due to persistent neuromuscular complaints, a noncontrast head CT and head MRI were done. These exams showed a faint hyperintensity in the left putamen and globus pallidus on T1-weighted MRI sequence (Fig. 1) and a hyperintensity on a T2-weighted scan in the left lentiform nucleus (Fig. 2). The neurology team was consulted consulted and nonketotic hyperglycemia hemichorea-hemiballismus syndrome was determined to be the patient’s diagnosis from symptoms and imaging findings as there was no diffusion restriction on MRI after receiving insulin as per basal bolus regimen in the emergency room. The patient’s insulin regimen was changed during her hospital stay, and tetrabenazine (50 mg/day) was also used to address her symptoms. Throughout admission, serum glucose level stayed high and fluctuated between 200 mg/dL and 400 mg/dL. The HbA1C level was 15.3%. On the fourth day of hospitalization, the patient was discharged with a suitable insulin regimen and tetrabenazine to treat choreiform symptoms. When she was discharged, both the frequency and the force of the uncontrollable arm motion had decreased but were still present. Observational notes from a subsequent follow-up visit 1 month later noted that the symptoms were still present, despite the fact that they were getting less severe and frequent. Repeat MRI was planned but not carried out due to financial constraints.

## Discussion

Hemichorea-hemiballismus (HC-HB) associated with nonketotic hyperglycemia (NKH) is a rare disorder that can occur in patients with poorly controlled diabetes. The estimated prevalence is around 1 per 100,000 diabetic patients, most commonly elderly Asian women with longstanding diabetes [1]. It is most frequently encountered in setting of acute ischemic or hemorrhagic stroke affecting contralateral basal ganglia, specifically the putamen [4]. The pathophysiology is not fully understood, but proposed mechanisms include vascular insufficiency, blood–brain barrier dysfunction, alterations in neurotransmitters such as gamma-aminobutyric acid (GABA), and enhanced dopaminergic activity [1, 2].

Our patient was an elderly woman with longstanding poorly controlled diabetes who presented with acute right-sided choreiform movements and NKH, aligning with characteristics seen in other reported cases [1, 5]. Neuroimaging revealed T1-weighted faint hypointensity in the left putamen and globus pallidus (Fig. 1) and a hypointensity on a T2-weighted FLAIR scan in the left lentiform nucleus (Fig. 2), a classic finding in diabetic striatopathy [3].

The rapid development of symptoms and imaging findings in the setting of NKH supports a metabolic rather than structural etiology. However, the underlying mechanisms are likely multifactorial. Small vessel disease, autoimmunity, and derangements in osmolarity and neurotransmitters such as gamma-aminobutyric acid (GABA) deficiency and alterations in dopaminergic activity may all contribute to the unique vulnerability of the basal ganglia in NKH [1, 6].

Despite the expectation that imaging abnormalities may resolve with glycemic correction, our patient will require close follow-up given the risk of recurrence, reported to be around 18% even after imaging normalization [1].

## Conclusion

This case highlights the importance of recognizing HC-HB as a rare presentation of NKH in poorly controlled diabetes. While anti-dopaminergic agents may be considered for symptom management, the mainstay of treatment is prompt correction of hyperglycemia.

## Data Availability

Not applicable.

## Abbreviations

NKH: Nonketotic hyperglycemia
HC-HB: Hemichorea-hemiballismus
CT: Computed tomography
MRI: Magnetic resonance imaging
ED: Emergency department
GABA: Gamma-aminobutyric acid
T1: T1-weighted MRI sequence
T2: T2-weighted MRI sequence
FLAIR: Fluid-attenuated inversion recovery MRI sequence
HbA1C: Glycated hemoglobin
